# Supplementary material for: The Splicing Factor SF2 Is Critical for Hyperproliferation and Survival in a TORC1-Dependent Model of Early Tumorigenesis in Drosophila
Source: Int J Mol Sci. 2020 Jun 24;21(12):4465. doi: 10.3390/ijms21124465 (PMC7352841; doi:10.3390/ijms21124465)
Supplement: Supplementary file 1 [file ijms-21-04465-s001.zip › Supplementary_figures.pdf]

Parniewska and Stocker

The splicing factor SF2 is critical for hyperproliferation and survival in a TORC1-dependent model of early tumorigenesis in *Drosophila*

Supplementary Figures S1-S11

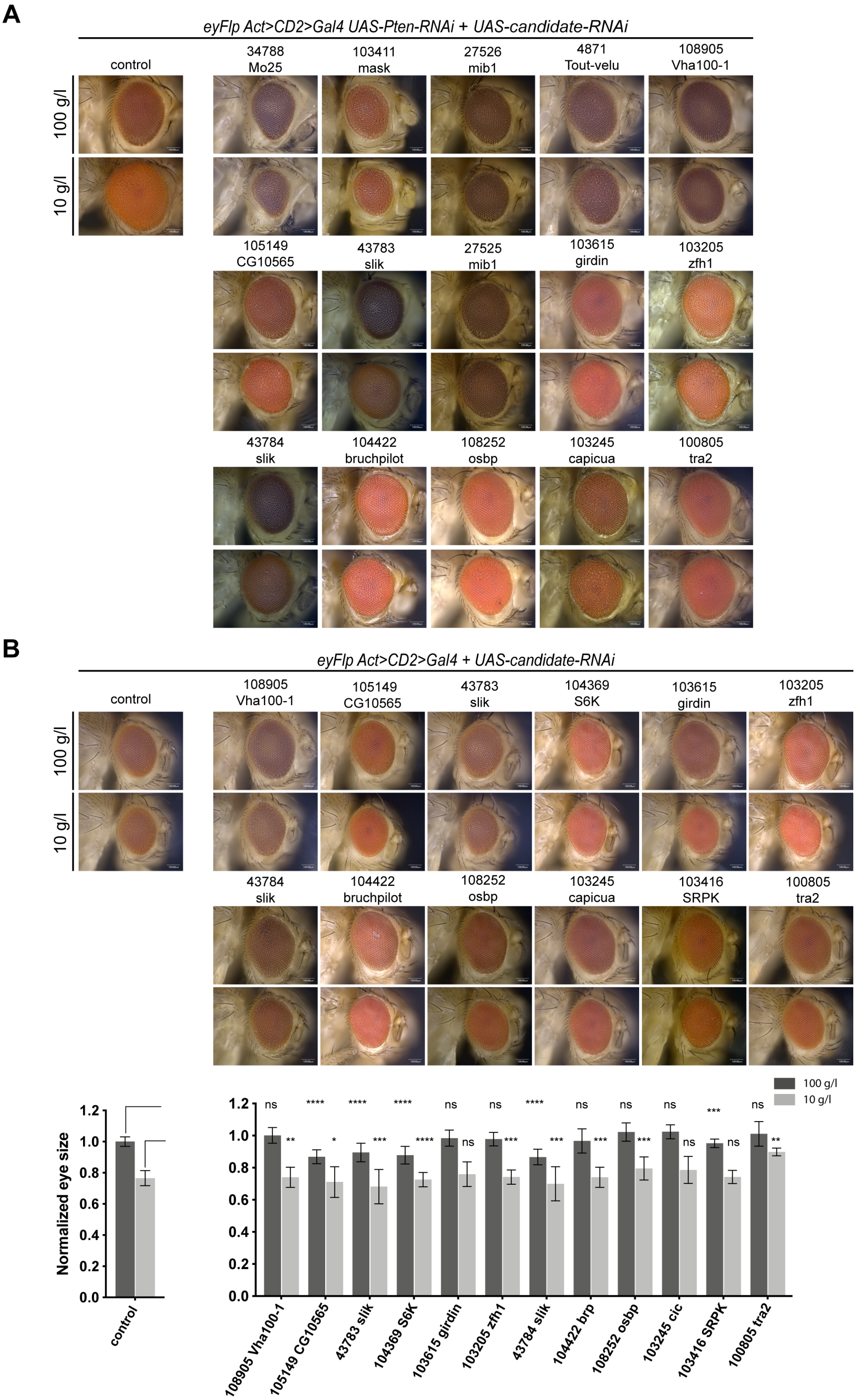
A
eyFlp Act>CD2>Gal4 UAS-Pten-RNAi + UAS-candidate-RNAi
control
34788 Mo25
103411 mask
27526 mib1
4871 Tout-velu
108905 Vha100-1
100 g/l
10 g/l
105149 CG10565
43783 slik
27525 mib1
103615 girdin
103205 zfh1
43784 slik
104422 bruchpilot
108252 osbp
103245 capicua
100805 tra2
B
eyFlp Act>CD2>Gal4 + UAS-candidate-RNAi
control
108905 Vha100-1
105149 CG10565
43783 slik
104369 S6K
103615 girdin
103205 zfh1
43784 slik
104422 bruchpilot
108252 osbp
103245 capicua
103416 SRPK
100805 tra2
100 g/l
10 g/l
Normalized eye size
control
108905 Vha100-1
105149 CG10565
43783 slik
104369 S6K
103615 girdin
103205 zfh1
43784 slik
104422 brp
108252 osbp
103245 cic
103416 SRPK
100805 tra2

Figure S1 Suppressors of the *Pten* overgrowth phenotype upon NR

A Eyes of suppressors not shown in Fig. 1 **B** Eyes and quantification of *eyFlp Act>CD2>Gal4* mediated knockdown of suppressor genes in control tissue. Only candidates not causing a severe reduction of control tissue were quantified. Since candidates were tested in several batches, the control represented on the left is a chosen representative. The eye size of each candidate is normalized to the control on normal conditions from the respective batch. Students t-test was used to test for significance of change between candidate knockdown and its control from the same nutrient condition, * $p < 0.05$, ** $p < 0.01$, *** $p < 0.001$, **** $p< 0.0001$, ns: not significant.

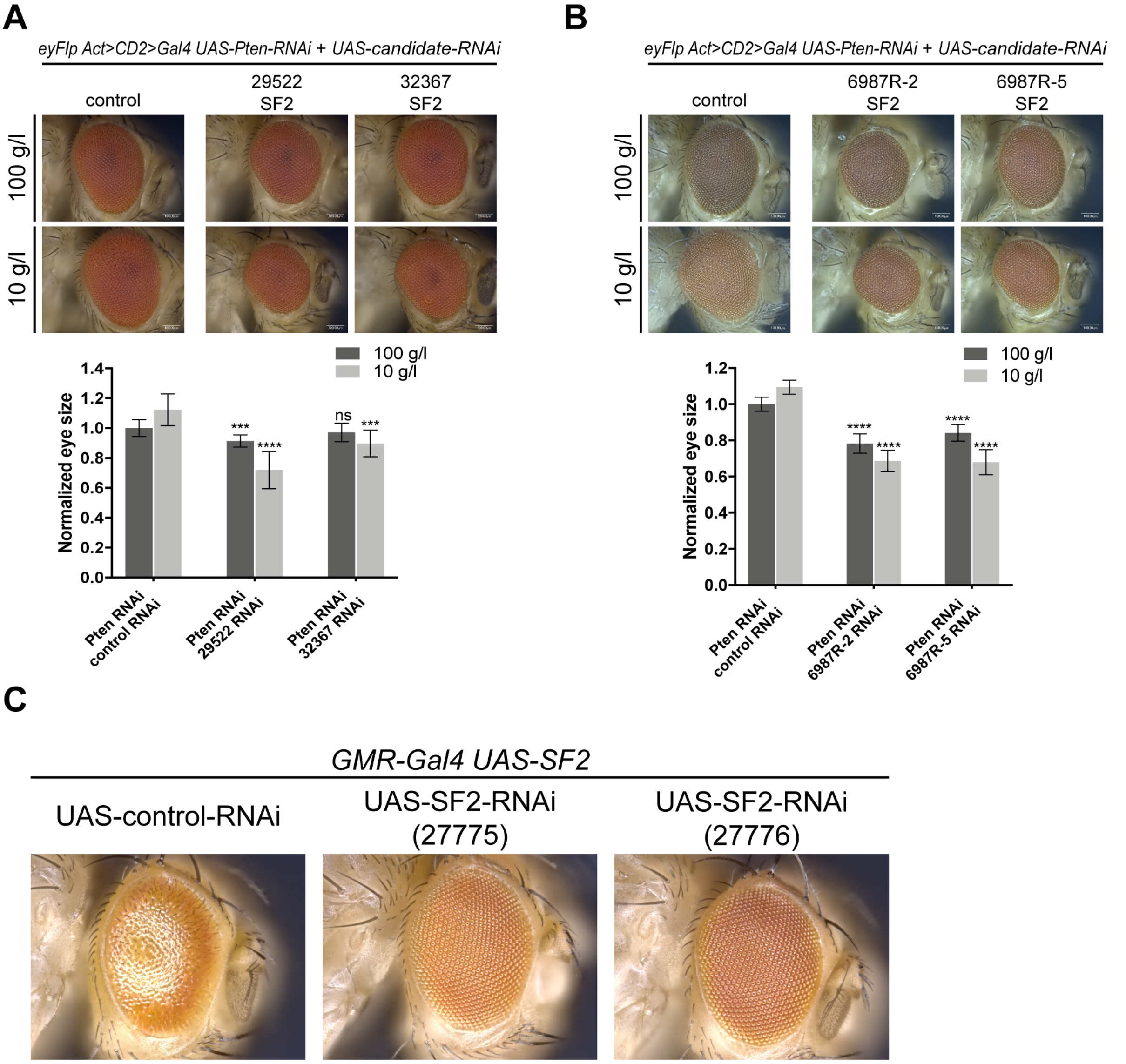

Figure S2 Validation of *SF2* RNAi lines

A Knockdown of *SF2* using TRiP (BDSC) and **B** NIG-Fly RNAi lines targeting independent sequences of *SF2.* **C** The two RNAi lines used in the screen (VDRC 27775 and 27776) completely suppress the malformations caused by *GMR-Gal4* mediated overexpression of *SF2*.

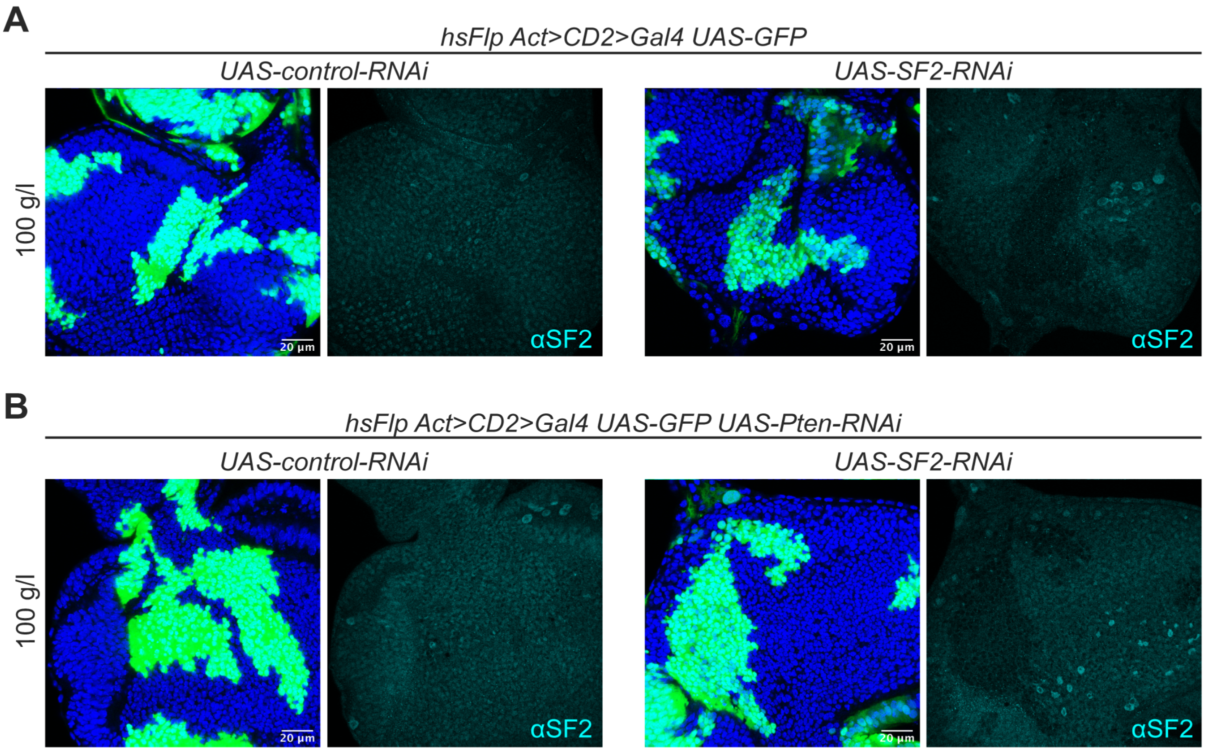

Figure S3 Analysis of *SF2* knockdown in clones

Clonal expression of *UAS-SF2-RNAi* was achieved by crossing it to *hsFlp; Act>CD2>Gal4 UAS-GFP* flies and inducing Flp expression by heat shock application. Knockdown of *SF2* using the VDRC line 27775 results in a decrease of SF2 on the protein level in both **A** control clones and **B** *Pten*-deficient clones as shown by SF2-specific antibody staining.

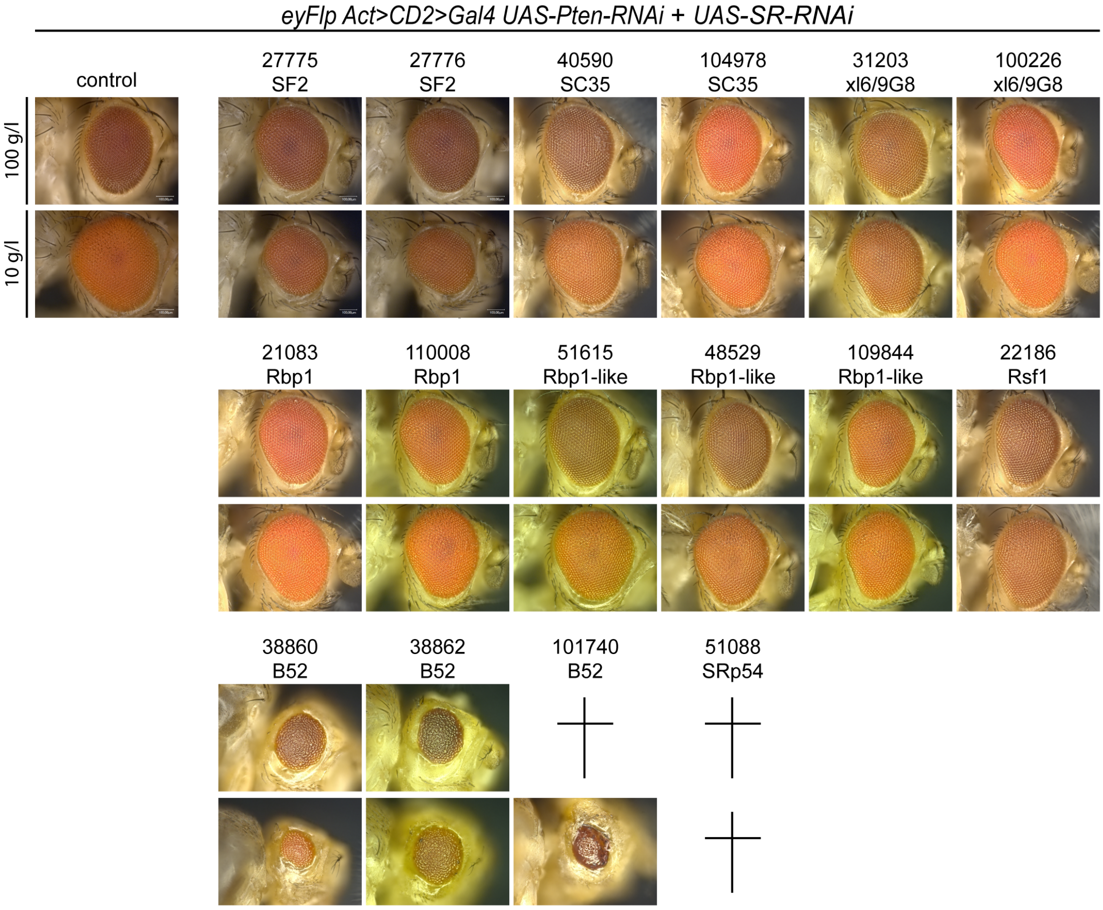

Figure S4 Eye-specific knockdowns of *Drosophila* SR-rich splicing factors

Phenotypes caused by knockdown of splicing factors from the *Drosophila* SR rich splicing factor family do not resemble the effect caused by *SF2* knockdown (VDRC lines 27775 and 27776). Most of the SR protein genes do not cause any change to the *Pten* overgrowth while B52 and SRp54 knockdowns result in severe malformations of the eye tissue.

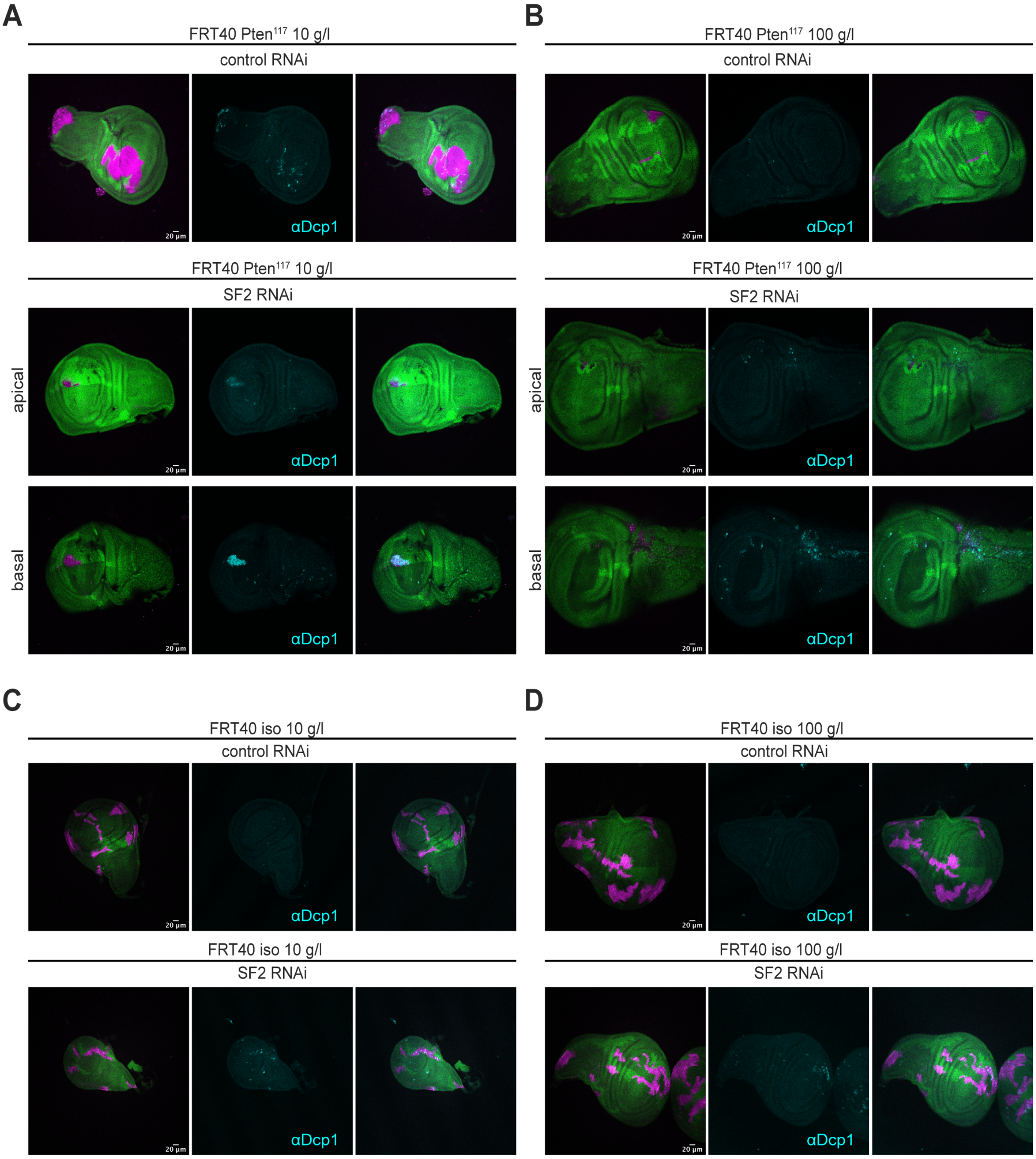

Figure S5 SF2 is required for the survival of *Pten* clones in the wing disc

Twin MARCM analysis of *SF2* knockdown in *Pten* clones in the wing disc. **A** Knockdown of *SF2* in clones of wing imaginal discs suppresses the growth of *Pten* clones upon NR with marked apoptosis at the basal side of the tissue. **B** *SF2* knockdown eliminates *Pten* clones also under normal conditions. **C** and **D** control (*iso)* clones are only mildly affected by *SF2* knockdown, displaying few apoptotic cells.

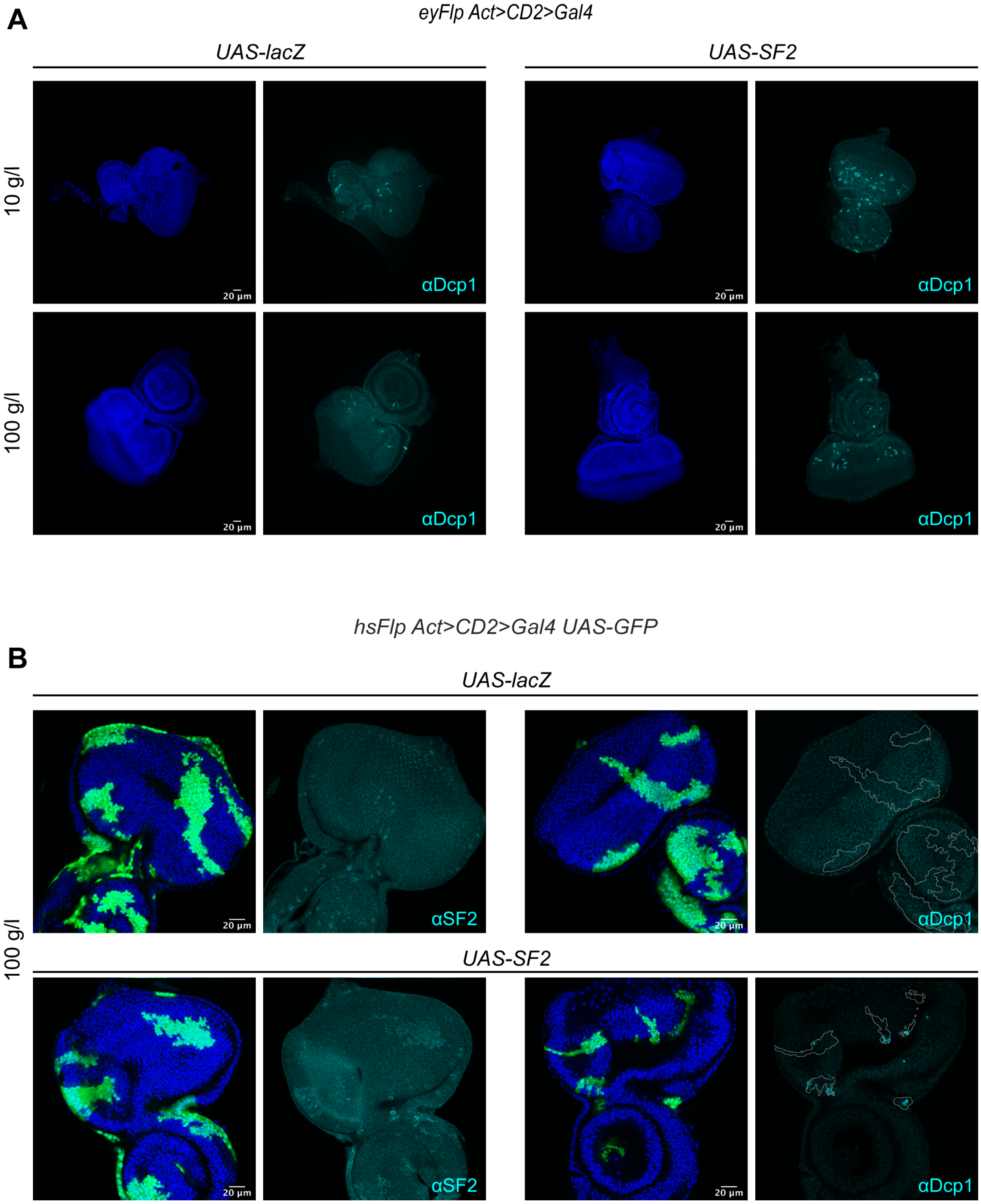

Figure S6 ***SF2*** **overexpression causes cell death**

A Overexpression of *SF2* in the entire eye-antenna imaginal disc (driven by *eyFlp Act>CD2>Gal4*) causes apoptosis that is more severe under NR conditions. **B** Clonal overexpression of *SF2* (achieved by *hsFlp Act>CD2>Gal4*) is lethal upon NR. Under normal feeding conditions, clonal *SF2* overexpression results in apoptosis of clones.

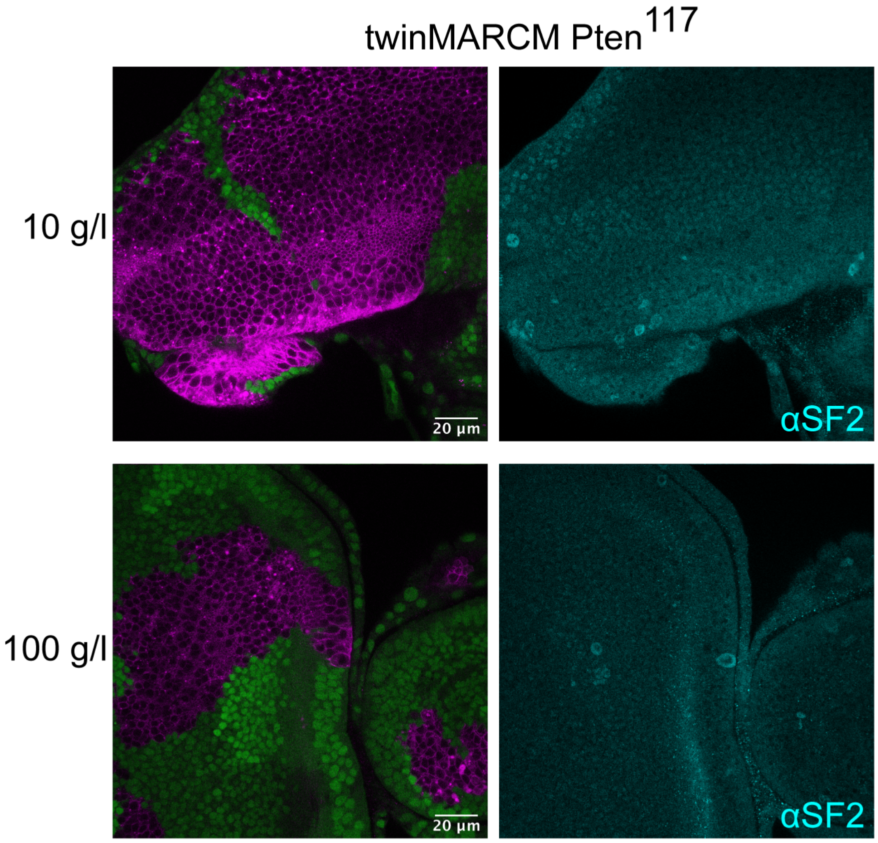

Figure S7 SF2 protein levels are not affected in *Pten* clones

SF2 protein levels do not change in clones of *Pten*^{117} mutant cells (generated using the twin MARCM system) under both normal and NR conditions as shown by an SF2-specific antibody staining.

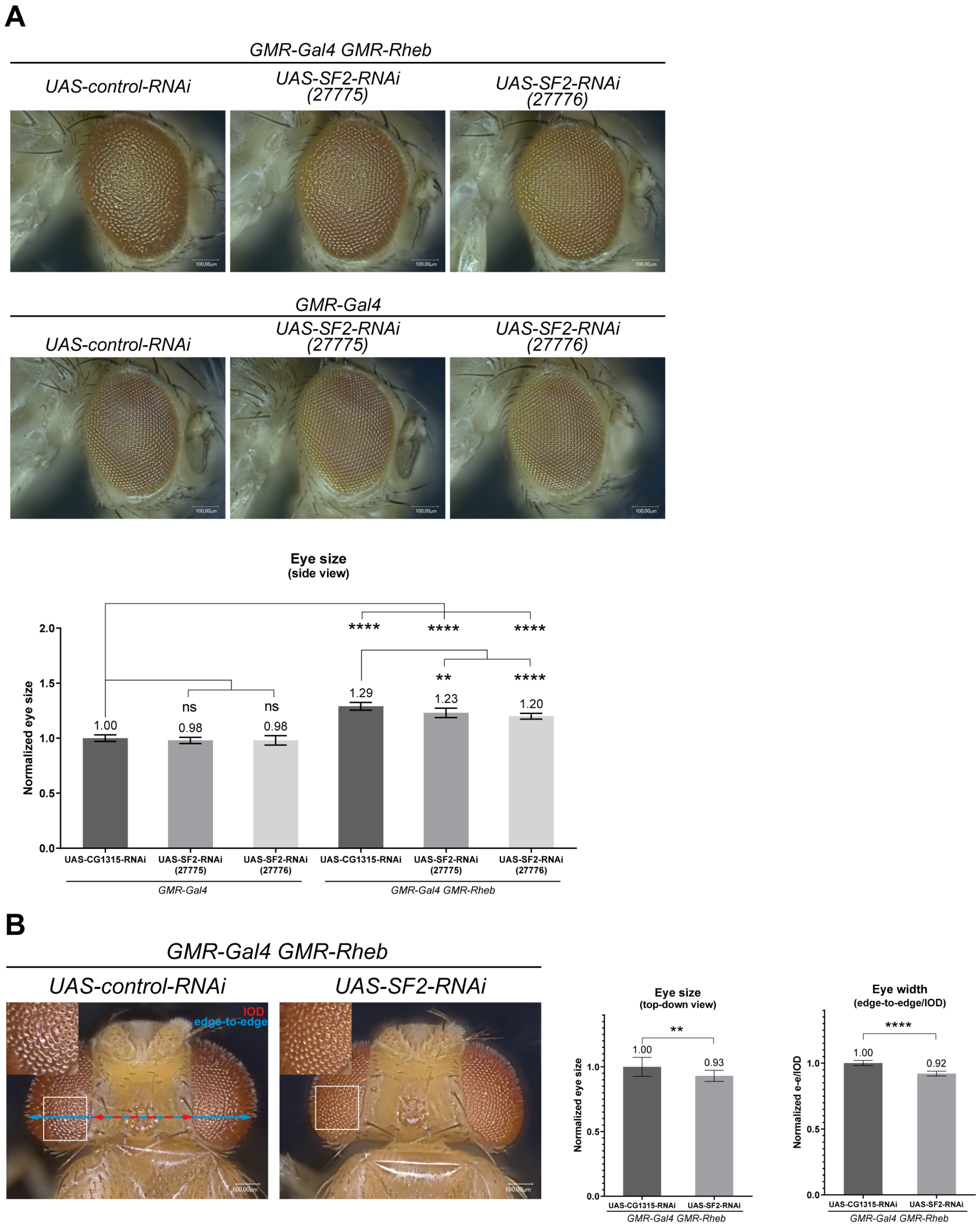

Figure S8 SF2 is required for Rheb-mediated overgrowth

SF2 knockdown partially suppresses the overgrowth phenotype caused by *Rheb* overexpression. Overexpression of Rheb was achieved by a *GMR-Rheb* transgene. *GMR-Gal4* was used to drive *SF2-RNAi.* **A** Side view showing suppression of the *GMR-Rheb* phenotype by the knockdown of *SF2*. The structural defects caused by *Rheb* overexpression are almost completely rescued. *SF2* knockdown does not affect eye size in a control background. **B** Quantification of eye size and eye width in top-down views. The insets show higher magnifications of the regions in the squares. Note that fewer ommatidia are fused upon *SF2* knockdown. IOC: interocular distance.

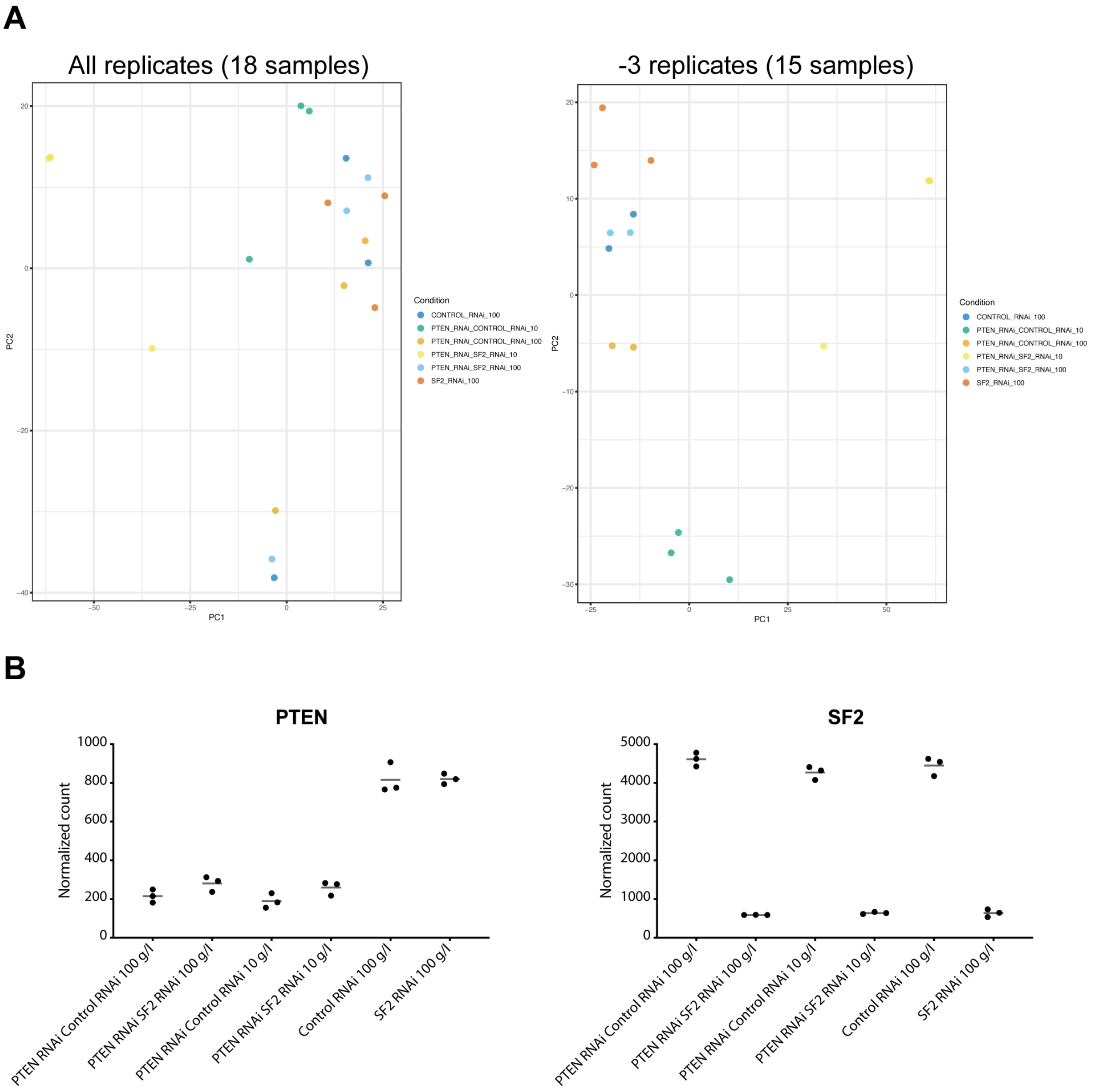

Figure S9 PCA analysis of replicates and efficiency of *Pten* and *SF2* knockdowns

A PCA analysis of all 18 replicates reveals that a separate cluster is formed by one replicate of: PTEN RNAi Control RNAi 100 g/l (orange), PTEN RNAi SF2 RNAi 100 g/l (light blue) and Control RNAi 100 g/l (dark blue). Although these replicates show the same extent of *Pten* and *SF2* knockdown compared to the other replicates (as evident in B), they were excluded them from edgeR and DEXSeq analyses. **B** *Pten* and *SF2* mRNA levels are efficiently reduced by the respective knockdowns.

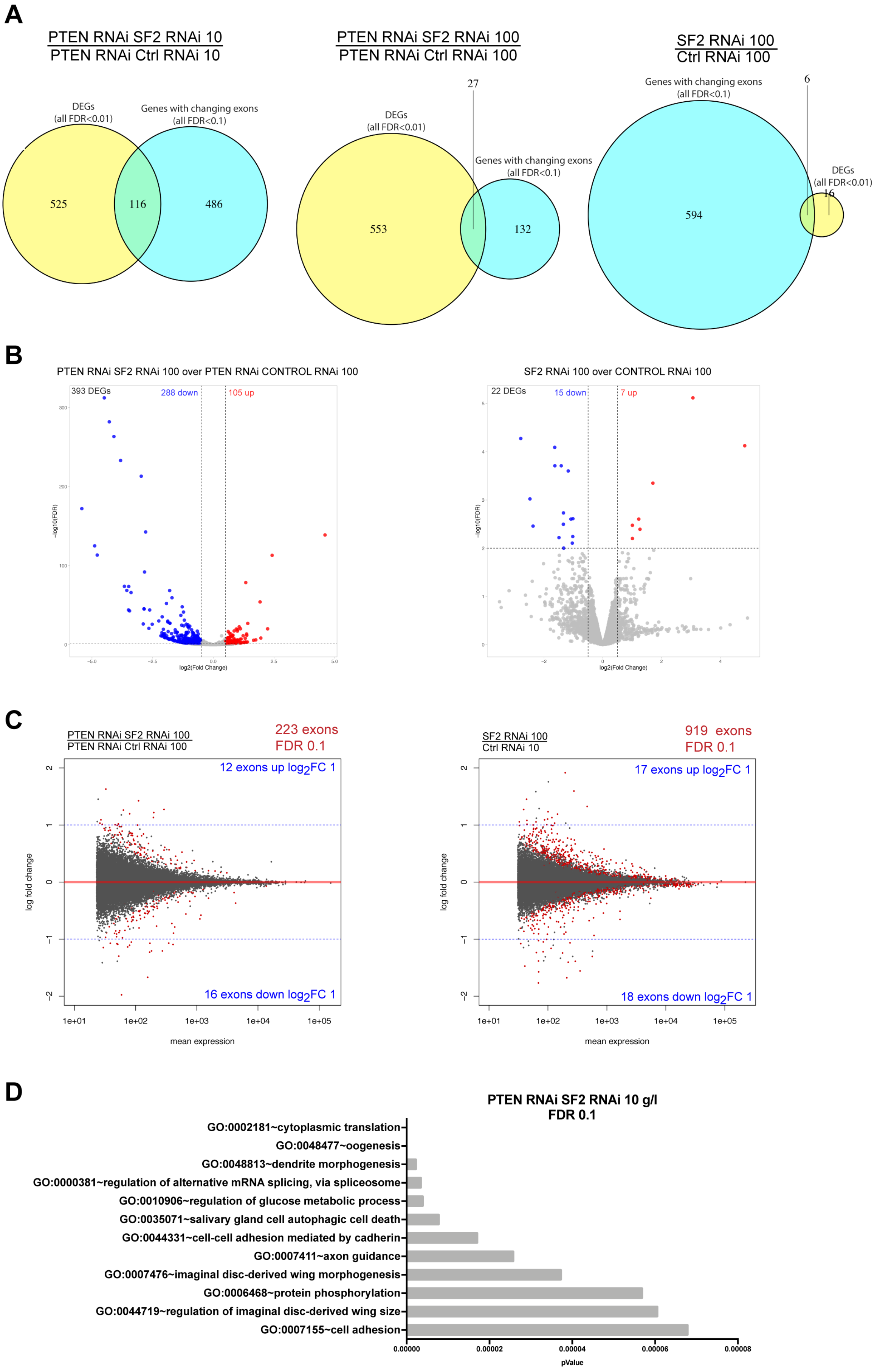
A
PTEN RNAi SF2 RNAi 10
PTEN RNAi Ctrl RNAi 10
DEGs (all FDR<0.01)
Genes with changing exons (all FDR<0.1)
525
116
486
PTEN RNAi SF2 RNAi 100
PTEN RNAi Ctrl RNAi 100
27
DEGs (all FDR<0.01)
Genes with changing exons (all FDR<0.1)
553
132
SF2 RNAi 100
Ctrl RNAi 100
Genes with changing exons (all FDR<0.1)
6
DEGs (all FDR<0.01)
594
16
B
PTEN RNAi SF2 RNAi 100 over PTEN RNAi CONTROL RNAi 100
393 DEGs
288 down
105 up
-log10(FDR)
log2(Fold Change)
SF2 RNAi 100 over CONTROL RNAi 100
22 DEGs
15 down
7 up
-log10(FDR)
log2(Fold Change)
C
PTEN RNAi SF2 RNAi 100
PTEN RNAi Ctrl RNAi 100
223 exons
FDR 0.1
12 exons up log2FC 1
16 exons down log2FC 1
log fold change
mean expression
SF2 RNAi 100
Ctrl RNAi 10
919 exons
FDR 0.1
17 exons up log2FC 1
18 exons down log2FC 1
log fold change
mean expression
D
PTEN RNAi SF2 RNAi 10 g/l
FDR 0.1
GO:0002181~cytoplasmic translation
GO:0048477~oogenesis
GO:0048813~dendrite morphogenesis
GO:0000381~regulation of alternative mRNA splicing, via spliceosome
GO:0010906~regulation of glucose metabolic process
GO:0035071~salivary gland cell autophagic cell death
GO:0044331~cell-cell adhesion mediated by cadherin
GO:0007411~axon guidance
GO:0007476~imaginal disc-derived wing morphogenesis
GO:0006468~protein phosphorylation
GO:0044719~regulation of imaginal disc-derived wing size
GO:0007155~cell adhesion
0.00000
0.00002
0.00004
0.00006
0.00008
pValue

Figure S10 DEGs and differentially expressed exons upon *SF2* knockdown

A Overlap between DEGs identified by edgeR and genes with changes in exon levels for the three two-group comparisons. For the comparisons, DEGs with FDR <0.01 and exons with FDR<0.1 were used. **B** DEGs in PTEN RNAi SF2 RNAi 100 g/l (left) and SF2 RNAi 100 g/l (right) detected by edgeR. **C** Exons changing in PTEN RNAi SF2 RNAi 100 g/l (left) and SF2 RNAi 100 g/l (right) detected by DEXSeq. **D** GO terms enrichment analysis of genes with exon changes in PTEN RNAi SF2 RNAi 10 g/l identified by DEXSeq.

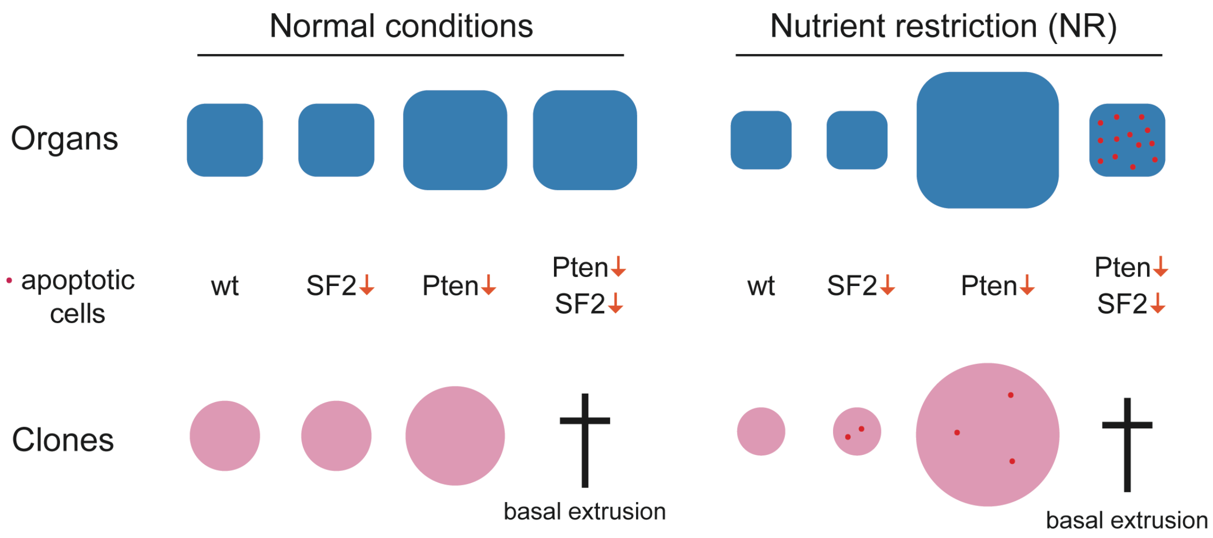

Figure S11 Schematic representation of SF2's effects on organs and clones

The effects of *SF2* knockdown on organs (eye discs and adult eyes) and clones are schematically depicted. Whereas *SF2* knockdown has no discernible effect on organ and clone size in a control background, it reduces organ size of *Pten* knockdown eyes specifically under NR conditions. In a clonal context, *Pten* mutant cells are fully dependent on SF2 function. *Pten* clones with an *SF2* knockdown are massively apoptotic and basally extruded from the epithelium (under all conditions).
